# Is there a relationship between sperm chromosome abnormalities and sperm morphology?

**DOI:** 10.1186/1477-7827-4-1

**Published:** 2006-01-25

**Authors:** Fei Sun, Evelyn Ko, Renée H Martin

**Affiliations:** 1 Department of Medical Genetics, Faculty of Medicine, University of Calgary, Calgary, Alberta T2N 4N1, Canada; 2 Department of Genetics, Alberta Children's Hospital, Calgary, Alberta T2T 5C7, Canada

## Abstract

This review explores the relationship between sperm chromosomal constitution and morphology. With the advent of techniques for obtaining information on the chromosome complements of spermatozoa, this relationship has been studied in fertile men and in men with a high frequency of chromosomal abnormalities. Using human sperm karyotype analysis, no relationship between sperm chromosome abnormalities and morphology was found in fertile men, translocation carriers or post-radiotherapy cancer patients. Fluorescence in situ hybridization (FISH) analysis has not generally revealed a specific association between morphologically abnormal sperm and sperm chromosome abnormalities, but has indicated that teratozoospermia, like other forms of abnormal semen profiles (aesthenozoospermia, oligozoospermia) is associated with a modest increase in the frequency of sperm chromosome abnormalities. However, FISH studies on some infertile men and mouse strains have suggested that certain types of morphologically abnormal spermatozoa, such as macrocephalic multitailed spermatozoa, are associated with a very significantly increased frequency of aneuploidy. Thus, there may be an association between sperm morphology and aneuploidy in infertile men with specific abnormalities.

## Review

Sperm topography is unique among the known cells and 3 major parts can be immediately distinguished: head, midpiece and tail. Normal spermatozoa exhibit an oval-shaped head with a regular outline and an acrosomal cap covering more than one-third of the head surface. The head length is between 3 and 5 μm, and its width ranges between 2 and 3 μm; the width is between one half and two thirds of the length. The midpiece is slender, less than one third of the width of the head, straight and regular in outline; it is aligned with the longitudinal axis of the head and is approximately 7 to 8 μm long. The tail is slender, uncoiled and should present a regular outline: it is at least 45 μm in length [1].

Abnormal sperm morphology is classified as defects in the head, midpiece or tail of the sperm [1]. Head defects include large, small, tapered, pyriform, round, and amorphous heads, heads with a small acrosomal area (<40% of the head area) and double heads, as well as any combination of these. Globozoospermia, where the sperm head appears small and round due to the failure of the acrosome to develop, is an example of a head defect. Midpiece defects include 'bent' neck (where the neck and tail form an angle of greater than 90% to the long axis of the head), asymmetrical insertion of the midpiece into the head, a thick or irregular midpiece, an abnormally thin midpiece (with no mitochondrial sheath), as well as any combination of these. Tail defects include short, multiple, hairpin, broken or bent (>90°) tails, tails of irregular width, coiled tails, as well as any combination of these.

A sperm is basically a package of streamlined genetic information. Intuitively, one might expect that a change in chromosome content is reflected by a change in the size of sperm – thus, people expect to see a relationship between sperm morphology and genetic abnormalities.

Aberrations in the genetic information of spermatozoa include numerical and structural chromosome abnormalities [2]. Numerical abnormalities include aneuploidies and polyploidies, and arise from a missing or extra chromosome(s) due to meiotic non-disjunction. Aneuploidies involve an autosome, a sex chromosome or both; polyploidies have a duplication of all chromosomes. Structural abnormalities include chromosome breaks, gaps, translocations, inversions, insertions, deletions and acentric fragments. The frequency of numerical chromosome abnormalities in sperm of fertile men is 1–2%, and the frequency of structural chromosome abnormalities in sperm varies from 7–14% [3].

It has been suggested that an assay for sperm morphology might prove useful as an initial screen in evaluating men at risk for an increased frequency of sperm chromosomal abnormalities [4]. The potential relationship between sperm morphology and chromosomal constitution has stimulated investigators to study this relationship in fertile men and in men with elevated frequencies of abnormal forms. The advent of techniques such as human sperm karyotyping and fluorescence in situ hybridization (FISH) analysis has allowed these relationships to be explored.

## Human sperm karyotype analysis

Using karyotype analysis of the human sperm pronucleus after hamster egg penetration, the association between the frequency of morphologically and chromosomally abnormal sperm was examined in fertile men [5], translocation carriers [6-8] and post-radiotherapy cancer patients [9-11]. This technique provides detailed information about each individual chromosome, permitting analysis of both numerical and structural abnormalities [12-14].

Martin and Rademaker [5] first evaluated the potential relationship between the frequency of chromosomally and morphologically abnormal sperm in thirty healthy fertile men of proven fertility, ranging in age from 22 to 55 years. The relationship between the proportion of sperm with chromosomal abnormalities (numerical, structural and total abnormalities [14,15]), the proportion of morphologically abnormal sperm (head, midpiece and tail defects [16]), and donor age was correlated using multiple regression analysis. There was no significant association between the proportions of morphologically and chromosomally abnormal sperm, when controlled for age. This was true for the total frequency of chromosomal abnormalities (*p *= 0.07), and for the frequency of numerical (*p *= 0.21) and structural (*p *= 0.32) abnormalities. It could be argued that no association between sperm morphology and chromosomal abnormalities was found because morphologically abnormal sperm did not penetrate the hamster eggs, and thus were not represented in human sperm karyotypes. However, no association was found between the proportions of hamster eggs penetrated and the proportion of morphologically abnormal sperm. These results provide indirect evidence that a morphological assay is not a good indication of chromosomal normalities in human sperm.

In men heterozygous for different translocations [6-8], the frequencies of sperm chromosomal abnormalities were very high, ranging from 33% to 92%, but an increased frequency of morphologically abnormal sperm was not observed. Similarly, the frequencies of abnormal sperm chromosome complements (both numerical and structural abnormalities) were significantly increased in post-radiotherapy cancer patients [9-11], with a range between 6~67%, but no increase in the frequency of morphologically abnormal sperm and no association between sperm morphology and testicular radiation doses were found.

Therefore, even in human populations at risk for a high frequency of sperm chromosome abnormalities (such as translocation carriers and cancer patients), sperm chromosomal abnormalities were not accompanied by an altered frequency of morphologically abnormal sperm.

## FISH studies on human sperm

In recent years, studies examining whether aberrant sperm morphology necessarily indicates abnormal chromosomal status in infertile men with elevated frequencies of abnormal forms have been carried out using FISH techniques. FISH analysis involves hybridization of chromosome-specific DNA probes labelled with fluorochromes to complementary DNA sequences on target chromosomes, followed by analysis of the bound probes under a fluorescence microscope. Characterized by its rapid application and easy accessibility, FISH analysis of decondensed sperm heads has proven to be an accurate and reliable method which allows the analysis of thousands of spermatozoa for chromosome aneuploidy in a relatively short period [17,18].

An elegant study was carried out by Celik-Ozenci et al. [19], in which sperm from fertile men were scored for aneuploidy using FISH, followed by a phase-contrast morphological evaluation of the same sperm. Their results showed that numerical chromosomal aberrations were present in sperm heads of all sizes and shapes, that some disomic and diploid sperm were completely normal-appearing, with a normally-sized, normally-shaped head and tail. Furthermore, sperm with normal chromosome constitutions also came in all shapes and sizes, some with abnormal morphologies. In this study of fertile men, morphology was deemed an unreliable method for the selection of sperm with a normal chromosomal content.

### FISH studies on infertile patients

Rives et al. [20] studied 50 infertile patients, including 2 asthenoteratozoospermia patients (<50% motility and <30% morphologically normal forms [1]), and found no correlation between sperm disomy frequency and motility or morphology for all analyzed chromosomes. Vegetti et al. [21] studied 32 infertile patients (7 patients with asthenoteratozoospermia, and 2 with teratozoospermia; all 9 had a normal karyotype) and found no significant association between sperm morphology and the frequency of sperm chromosomal aneuploidy. Viville et al. [22] examined the chromosomal content of spermatozoa from 3 patients affected with either globozoospermia, shortened flagella syndrome, or spermatozoa with an abnormal acrosome. The absence of any significant increase in the aneuploidy rate suggested that there was no significant relationship between the frequency of numerical chromosomal abnormalities and these specific morphological abnormalities. Although not controlled for sperm concentration, these studies indicated that in general, there is no specific correlation between the frequency of aneuploidy and sperm morphology in infertile men [20-22]. This lack of a relationship between sperm morphology and an aneuploid chromosomal constitution agrees with the data obtained by sperm karyotyping analysis using human spermatozoa-hamster egg fusion techniques [5] and after injection of human spermatozoa from semen samples with a high incidence of amorphous, round and elongated sperm heads into mouse oocytes [23]. Although there was no increase in numerical chromosomal aberrations in the latter study, Lee et al. [23] did find an increased frequency of structural chromosome abnormalities such as chromosome/chromatid fragments, dicentric and ring chromosomes.

### Infertile patients with teratozoospermia

A modest increase in the frequency of sperm chromosome abnormalities has been found in infertile men with teratozoospermia (<14% normal forms) who had a normal sperm concentration and karyotype. Gole et al. [24] found that sex chromosome disomy in 8 men with teratozoospermia was increased about 4-fold compared to normozoospermic groups. Similarly, sperm chromosomes in men with teratozoospermia and asthenoteratozoospermia had a 2~3 fold increase in numerical abnormalities compared to normal controls [25-28]. Ushijima et al. [29] reported that the incidence of sex chromosome abnormalities increased significantly with an increase in the percentage of morphologically abnormal spermatozoa in eight oligoasthenoteratozoospermia (OAT) patients. These modest increases in sperm aneuploidy suggest that teratozoospermia (like abnormal motility and low concentration) is a marker of abnormal spermatogenesis which is associated with elevated sperm aneuploidy in general.

### Globozoospermia

Similarly, a modest increase in the frequency of sex chromosome aneuploidy (2–3 times that in controls) has been found in the sperm of patients with globozoospermia (round-headed sperm) [30-35]. Globozoospermia is a very rare condition observed in <1% of infertile patients, where the major morphological anomaly is the absence of an acrosomal cap in sperm. There is also some suggestion that globozoospermia may be associated with abnormalities in chromatin structure since an increased frequency of sperm with DNA strand breaks (using TUNEL assays) has been found in these patients [33], but this has not been observed in all studies [36]. Mouse models may permit exploration of the mechanisms leading to the abnormalities in this disorder, as a mouse knockout for the casein kinase catalytic subunit gene (which causes globozoospermia and sterility in mice) has been described [37].

### Macrocephalic, multinucleated and multiflagellate sperm

In one specific group, a significant association between sperm morphology and sperm chromosome content has been found: in teratozoospermic men with a high percentage of macrocephalic, multinucleated and multiflagellate sperm, very high frequencies of aneuploid and polyploid sperm have been reported. Viville et al. [22] observed a very high frequency of aneuploidy (67%) in a patient with 64% macrocephalic spermatozoa. Benzacken et al. [38] reported an OAT patient who had 100% macrocephalic sperm heads, 38% with an irregular acrosomal cap and 72% with 2–5 flagella. FISH analysis of 1,148 spermatozoa showed 100% chromosomally abnormal sperm: 21.6% were diploid, 62.4% triploid, 13.3% quadriploid and 2.7% hyperploid. In an OAT patient who had 100% sperm with macrocephalic heads, one to three tails and an absence of an acrosomal cap, In't Veld et al. [39] reported that normal haploid sperm were virtually absent (<2%), and abnormalities included 40% diploid and 24% triploid sperm. Another report [40] detailed high frequencies (>50%) of chromosome 18, X and Y disomies in sperm from 3 men with 100% teratozoospermia: double-headed sperm (>20% incidence in all three patients), large-headed multinucleated sperm (>50%) and multiple tail deformities (>50%). Devillard et al. [41] reported more than 50% sperm aneuploidy for chromosomes 1 and 18 in three patients with 100% large-headed multiflagellate sperm. Bernardini et al. [42] reported 20% disomy and 10% diploidy in 6 infertile men with macrocephalic or two-tailed spermatozoa. Thus there is consistent evidence for a relationship between these specific morphological sperm abnormalities and abnormal chromosomal constitutions.

## Mouse studies on sperm morphology and chromosome contents

Specific mouse strains with a high incidence of morphologically abnormal sperm have been studied to examine the relationship between sperm morphology and chromosome constitution in two studies [43,44].

Kishikawa et al. [43] microinjected individual mouse sperm with either normal or abnormal morphology into enucleated mouse oocytes and assessed the relationship between sperm morphology and karyotypes. BALB/c male mice, known to produce an unusually high proportion of morphologically abnormal spermatozoa (75%) [45], were used as sperm donors in this study. Abnormal karyotypes, most with structural breaks and exchanges, were found in 37% of eggs injected with morphologically-abnormal sperm (misshapen sperm heads: collapsed and triangular) – significantly higher than in those injected with normal sperm heads (mean 18%, although this, in itself, is an extraordinarily high frequency). These results suggest a possible association between morphologically abnormal BALB/c spermatozoa and structural chromosome abnormalities.

The PL/J mouse strain also has a high frequency of sperm with abnormal head morphology (30%) [46], including enlarged heads, narrow and elongated heads and no head (mitochondrial drop) [44,46]. Using FISH methods, Pyle and Handel [44] reported that the frequency of hyperhaploidy (mean 3.4%) in PL/J mouse sperm was significantly higher than that in control B6 mice (mean 0.3%), which might be attributed to strain differences. Numerous meiotic abnormalities contributed to sperm aneuploidy in the PL/J mice. Immunolocalization analysis of the synaptonemal complex (SC) during sperm meiotic prophase showed chromosome asynapsis [47], while analysis of the frequencies of MLH1 foci on SCs in pachytene spermatocytes and direct counts of chiasmata visualized in metaphase I indicated a reduction in crossovers [48,49]. During the first meiotic division, roughly one-third of PL/J mice spermatocytes exhibited aberrant spindle morphology, with abnormalities including monopolar spindles, split spindle poles, incomplete spindle formation and centrosomal abnormalities. These studies indicate that in PL/J mice, numerous meiotic abnormalities predispose to sperm aneuploidy and possibly abnormal sperm head morphology.

## Conclusion

Human sperm karyotype studies do not suggest any relationship between morphology and numerical chromosomal abnormalities in sperm. FISH studies on teratozoospermic men with a normal sperm concentration demonstrate a small, but significant, increased frequency of sperm aneuploidies for some chromosomes. Men with a specific type of teratozoospermia (macrocephalic multiflagellate sperm) have a very high frequency of aneuploid and polyploid sperm.
